# New York Pathological Society

**Published:** 1856-08

**Authors:** 


### ECLECTIC DEPARTMENT,

### AND SPIRIT OF THE MEDICAL PERIODICAL PRESS.

---

*New York Pathological Society.* Reported by E. LEE JONES, M. D., Secretary.

REGULAR MEETING, Feb. 13, 1856.

*Chylous Urine, Microscopic Examination.*—Dr. Alonzo Clark exhibited a *small vial of urine*, which he was informed was passed in its present milky condition; from its appearance, he thought it to contain urate of ammonia, but on subjecting it to examination by the microscope, an immense number of extremely minute granules, and a few oil globules were observed, and it was noticed that a large number of vibriones were present, formed unusually early, as the urine was kept in a cold room, and examined the morning after it was voided. The milkiness is caused by the little granular masses diffused throughout the fluid. In the other vial is a mixture of urine two parts, and ether one part; the milkiness is entirely removed by the ether, and it is seen to have separated into two distinct layers, the upper one is transparent, the lower of an amber color, and gelatinous consistence; a large amount of albumen is also present. This specimen was passed by a clerk in a liquor store, who was in the habit of freely drinking gin—four or five days before the urine in the vial was passed, he is informed that it was clear and natural in appearance—his health has not suffered. He would inform himself more particularly in regard to the case, and report at a future meeting of the society.

*Aneurism of Aorta leading into Pleural Cavity.*—Dr. George T. Elliot, presented an *aneurism* of the *aorta* removed from a woman, a patient of Bellevue Hospital, 30 years old. When received, she complained of pain over the sacrum, and a general debility, nothing else seemed to be present, —tonics were administered. Suddenly she was seized with a sharp pain over the heart and left shoulder, and great prostration. The house physician was summoned, and on examination discovered dullness over left side, and absence of the respiratory murmur; in a few minutes she died.

Post-mortem examination disclosed an aneurism of the aorta, which had discharged its contents into the left pleural cavity, where was found a pint and a half of clotted blood.

*Encysted Kidneys.*—Dr. Van Buren presented for Dr. Gentry, of Bellevue Hospital, a well-marked and beautiful specimen of *encysted disease* of the *kidneys*, weighing 14 ounces. When first removed, the surface of both was entirely covered with cysts, varying in size, some containing an ounce of fluid,—in some of a yellow glairy consistence, in others, of grumous character. The microscope showed nothing peculiar except granular material and a few oil globules. On section, the integrity of the cortical and medullary tissue is seen to remain. The medullary structure was carefully examined with a view of ascertaining if the cysts were due to a dilatation of uriniferous tubuli; the inference derived from the examination seems not to warrant this conclusion.

Dr. Clark inquired if there was any dilatation of the investment of the Malpighian bodies.

Dr. Van Buren replied that they were not examined.

Dr. Clark observed that the opinion is entertained by some, that the disease depends on a dilatation of the uriniferous tubes investing and reflected over the Malpighian bodies; and that this is the seat of the degeneration, and the effusion occurs on the attached surface.

Dr. Van Buren stated that it was not clearly ascertained if the sheath was so reflected.

Dr. Clark had himself injected the uriniferous tubes with a solution of indigo, and stained the structure, which looked as if it might be that investing membrane.

*Aneurism of the Internal Ilia.*—Dr. Van Buren next presented a specimen rather of surgical than pathological interest,—an *aneurismal sac* of the *internal iliac* artery, which was removed from a patient of St. Vincent's Hospital. At the time of admission his system was much reduced, and shattered by the use of opium and stimulants. On examination, an immense pulsating tumor was seen situated both above and below Poupart's ligament. Over its most prominent part was a black eschar, which looked as if it might burst at any moment. On consultation, it was concluded that an attempt to tie the artery should be made. A large incision was made; and, carefully pushing up the peritoneum, an artery was felt, apparently healthy in structure, and supposed to be the external iliac. A ligature was passed around it, with the effect of controlling the pulsation. The next day he was doing well; but in three days active inflammation of the tumor ensued, and he sank rapidly, and died on the fifth day.

Post-mortem examination disclosed the ligature around the primitive iliac, an inch above its bifurcation into external and internal; a well-formed clot existed in the internal iliac. No peritonitis. Extensive suppuration had occurred in and around the sac.

*Ovarian Tumor.*—Dr. E. A. Peaslee presented an *encysted ovarian tumor*, weighing 45 pounds, removed from a lady aged 41, who first observed a tumor in the left iliac region between three and four years ago, which has gradually increased up to the present time (Feb. '56) with slight fluctuations in size. It had from the first been regarded as *ovarian.* The patient was first seen by Dr. P., in May, 1855; she wishing to obtain his opinion in regard to the propriety of an operation for its removal by the large abdominal section. On examination, he found the abdominal circumference to be 47 inches, the walls of the abdomen so tense that he could not decide whether the mass consisted of many or few distinct sacs. The general condition of the patient was so low, that he did not for a moment entertain the idea of an operation, and gave his opinion accordingly.

He did not again see her till the 25th of last month (Jan. '56) when he was again requested to remove the mass. To his surprise, her condition had much improved since May, '55 (though she had failed during the past summer); and, though the tumor had risen somewhat higher in the epigastrium, her circumference was but forty-eight inches. Appetite pretty good; respiration somewhat hurried, though, when sitting or lying quiet, there was no dyspnœa. Bowels regular, action of kidneys rather free. He did not, however, *advise* the operation of ovariotomy; though to her inquiry whether she was apparently in as good a general condition as the two persons on whom he had operated successfully, he was obliged to reply in the affirmative; and, moreover, that it was impossible to ascertain whether the mass was adherent or not, without previously evacuating the sacs, by tapping to such an extent as to admit a more exact examination; and that he could not express any opinion in favor of ovariotomy, without previously tapping her; and if in doing this, he found the mass extensively adherent, or could not decide it was *not* adherent, in that event, he would not entertain the idea of an operation. He did not advise the tapping even; since, though he regarded this operation as hardly dangerous in any degree, she was informed that the mass might be made up of very many small sacs, and without a single large one, and in that case she would be disappointed, and *he* should not arrive at any positive result as to the adhesion or non-adhesion of the tumor. The patient had a decided aversion to being tapped, unless she was assured that ovariotomy would follow; since a sister, who had being tapped a few years since, for the same disease, died a week after of peritonitis, and because she supposed, if once tapped, a repetition of the operation would be frequently necessary. After a deliberation of five days on the subject, the patient again sent for Dr. P., and informed him she had decided to be tapped, as preliminary to the decision of the question, whether he would perform ovariotomy or not. The operation was performed in the usual way, on the 4th inst., assisted by Dr. Ranney, of 23d street. Her condition was good. Fluctuation indicated the existence of a distinct sac of considerable size, in and below the umbilical region, and another higher up. The former was at once reached by the trocar, and six pints of clear and highly gelatinous fluid (to the sense of touch) evacuated; and on partially withdrawing the canula, two pints more of a milky fluid were withdrawn, evidently from another sac, which had been traversed by the instrument, while on its way to the larger one. On changing the direction of the canula to penetrate another sac, a few drops of venous blood flowed through the instrument. This, he thought, proceeded from a minute vessel on the interior of the sac, which had been punctured, as he had seen the same thing before. Several smaller sacs were then punctured, and on withdrawing the canula as before, a few drops of venous blood again appeared. Fearing this might escape through the puncture in the sac into the cavity of the peritoneum, he waited until the dropping entirely ceased, and then withdrew the canula. With a curved trocar, other more distant sacs were evacuated. The mass now seemed to be composed of small cysts, and it seemed impossible to reduce the tumor much more. Further attempt was therefore discontinued. More blood now flowed through the canula; he waited till all oozing ceased, before finally withdrawing the instrument. Fifteen pounds of fluid had been withdrawn. The patient was fatigued by the prolonged operation, and depressed in mind from the fact that the operation must fail to demonstrate the adherence or non-adherence of the tumor; but with the exception of some faintness and sickness of the stomach, nothing worthy of mention occurred. The tumor could be slightly moved below the umbilicus, but not at all at the upper part; the idea of the operation of ovariotomy was therefore abandoned. The next morning before 10 o'clock, he was requested to visit the patient in haste, as she seemed to be sinking; before he arrived she was dead. She had passed a tolerably comfortable night, with sickness of the stomach at times, but presented no grave symptoms till 8 o'clock, when her expression changed, and she became restless, and died before 10.

Post-mortem examination, six and a half hours after death. Some bloody serum had escaped from the puncture through the abdominal walls. On cutting through the latter on the median line, a thick and very vascular membrane was found intervening between the parietal peritoneum and the ovarian mass; and a layer of bloody serum was seen between this and the mass, one or two inches deep. This membrane was found to cover over the whole tumor anteriorly and laterally, like an apron, it being also adherent to the tumor on both sides, as well as to the pelvis and the lower portion of the tumor. On further examination, the membrane just described was found to be the omentum major; and hæmorrhage had proceeded from a small vein, which had been punctured in penetrating it to reach the first sac. It had become so thick and firm, as well as vascular, by constant pressure and the motions of the tumor, that he had mistaken it for the wall of the sac first punctured, and the blood, which, during the operation, he supposed had flowed from the inner wall of the sac, had really flowed from the membrane just mentioned, into the cavity formed by the adhesions before specified, between itself and the diseased mass. But little bloody serum had escaped into the cavity of the peritoneum; and it was judged that not more than eight or ten ounces had been lost in all. The tumor (which was shown) was found extensively adherent to its upper and lateral portion, not so much so below the umbilicus. Its removal would not have been attempted during life, had it been exposed to view for that purpose, by any judicious surgeon. It was found to consist of an immense number of small sacs, as you perceive; and weighed forty-five pounds, making sixty pounds in all before the operation of paracentesis. It was chiefly developed from the left ovary; and both Fallopian tubes were closed up and distended with a putty-like substance, in which broken-up epithelial cells predominate.

It may be proper here to remark, that though a married lady for several years past, she had never been pregnant; menstruation had been regular till within the last one and a half years. Dr. P. observed that the hæmorrhage must be regarded as the "causa sine qua non" of death in this case. That is, had no hæmorrhage occurred, death might not have taken place in any immediate connection with the operation. A quantity of blood between the omentum and the tumor, with a small amount also in the peritoneal cavity, must have in a few days led to a fatal result; but in accounting for a death occurring within sixteen and a-half hours after the operation, and where the amount of blood lost was so small, we should doubtless also take into consideration the exhaustion from the operation, and especially the mental shock produced by the knowledge that the operation had led to no positive result in diagnosis, and that therefore nothing further would be done.

The *source* of the *hæmorrhage* was, as far as he knew, *peculiar*. Branches of the internal epigastric artery have sometimes been wounded; the bladder has been wounded; the uterus, happening to lie in front of the tumor, has also been punctured; and one of the Fallopian tubes, also happening to be stretched over it in front, has been transfixed. But he has never heard of the greater omentum being injured by a puncture, at a point usually regarded as the safest, —half way between the pubis and the umbilicus. Indeed, in all ordinary circumstances, where the abdomen is largely distended, it is impossible that the omentum should extend to this point. For it is not long enough, naturally, to extend even to the umbilicus in a case like this, even though it originally fall into the pelvis: and, moreover, it is uniformly, as far as he is aware, pushed up by the tumor during its development from below, and is generally found somewhat folded, and not reaching more than half the distance from the stomach to the umbilicus. In this case, the omentum was not less than two and a half feet long, as the specimen will show, since it completely covered the tumor anteriorly and laterally. And since, had it been free at its lower extremity at the time the tumor first began to grow, the latter would doubtless have merely lifted it up as is usual. Dr. P. inferred that the omentum had become adherent to some portion of the pelvic peritoneum before the tumor began to be developed. Thus the tumor grew upward behind the omentum, which thus was expanded over the whole length of the tumor.

Finally, the whole extent of the omentum was equally vascular; and had the puncture been made at any other point, there is no reason for believing that the hæmorrhage would have been less than that which actually occurred.

*Caries of Elbow-Joint—Exsection.*—Dr. A. C. Post presented the extremities of the bone of the elbow-joint, removed by the operation of exsection, for caries, from a boy fourteen years old. The patient was progressing well, and he anticipated his having a serviceable limb. Dr. P. again exhibited the specimen of *eburnation* of the *femur*, shown to members two meetings since. He had macerated the bone, and it is observed that its inferior portion is in a carious condition; above that, there is necrosis of the shaft; and within the medullary cavity is a sequestrum, which, though movable, cannot be withdrawn, being retained by two small holes, each of them having a peg of bone, passing down to the sequestrum. The specimen was mainly interesting from the fact of its presenting anchylosis, caries, necrosis and eburnation.

*Valvular Disease, &c., &c.*—Dr. O'Rorke exhibited a specimen of *valvular disease of the heart* and *dilation of the ascending aorta, with constriction of that vessel*, and gave the following history of the case:

Caroline L., aged thirty-six, born in England, came under observation in May, 1855. She complained of distressing pain in cardiac region, palpitation, and vertigo, with frequent attacks of syncope following agitation or excitement, especially that of the sexual organs. She had also chronic rheumatism in most of her joints; had acute rheumatism several years ago. She was accustomed to hard work, was very excitable, and was ænemic and very much emaciated; but never had œdema of the extremities. Her pulse was equal in both wrists, small, weak, and jerking or receding; there was a thrill communicated to both the carotids. She had increased dullness over the precordial region, but the rest of the chest was resonant on percussion, except a small space on the right of the sternum and between the third rib and the clavicle, where there was slight pulsation. The heart's action was attended with strong impulse. The apex struck the chest one inch and a quarter to the outside at the nipple, and between the sixth and seventh ribs. Rhythm was natural. Over the heart was heard a loud, systolic, bellow's murmur, most distinct over the apex. Over the aorta valves was a double murmur, the systolic being loud, coarse, and passing up the aorta, and heard distinctly in the second intercostal space to the right of sternum. Also at the top of sternum it was quite audible between the upper and middle third of the scapula on each side of the spine; and between the spine and the lower angle of the scapula; it was more distinct on the left side than on the right. There was a harsh, sharp murmur at the commencement of the diastole. The diagnosis was *hypertrophy of the heart*, accompanied by *mitral regurgitation*, with *aortic obstruction and regurgitation with aneurism of ascending aorta.* The treatment was tonic and sedative in its character, each new prescription being followed by marked relief. Still her paroxysms of pain, palpitation, and syncope would return at short intervals, she thinking that each one would end in death. She was out on business on Monday, Feb. 11th, 1856, apparently as well as usual. On the morning of Wednesday, Feb. 13th, she had an attack of pain, palpitation, &c., when she suddenly expired.

Post-mortem examination was made eight hours after death, in the presence of Dr. Leaming (to whom I am indebted for the opportunity of doing so), Drs. Tucker and Clark.

On opening the chest, the lungs did not collapse. They filled the chest completely, and were healthy, except in being slightly emphysematous. There were no traces of old pleurisy. The pericardium contained from six to eight ounces of clear serum, and the right pleura twice that amount. The abdominal organs were healthy. There was also hypertrophy of the left ventricle of the heart, the walls being twelve lines thick. The ventricular cavity, however, was not dilated, and the right side was not at all increased in size. The mitral orifice was contracted to a little more than half its normal dimensions, being capable of admitting only the end of one finger; but it was quite smooth. The anterior mitral valve, or that between the mitral and aortic orifice, was thickened and somewhat shortened, though not at all roughened; the other seemed to be absent; being so much contracted that the chordæ tendineæ appeared attached to the tendinous margin of the auriculo-ventricular opening. The aortic or semilunar valves were thickened, contracted, and adherent to each other at their sides, so as to stand out, forming a ring around the orifice, and of course obstructing the passage of the blood from the ventricle; yet in their constriction they failed to meet in the centre, and admitted of considerable regurgitation. The circumference of the ascending aorta, one inch above the free margin of the semilunar valves, measured three inches and eight lines. Immediately outside the attachment of the remains of the ductus arteriosus, the descending aorta, instead of descending by a gradual curve or arch, as usual, descended abruptly, thereby forming an angle. At this point the under wall or coats of the artery became, as it were, folded inward or upward; forming a sulcu[illegible] one and a half or two lines deep on the concave or lower wall, and of course, projecting into the vessel. Upon the projecting angle thus formed internally, the internal and middle coats were destroyed for about four lines transversely, and about one line longitudinally, and the internal and middle coats dissected up for one and a half or two lines on the distal side of the fissure thus formed by the destruction of the coats of the vessel. At this point the circumference of the artery was only one inch and seven and a half lines, being *seven lines* less than it should have been when compared with the mean circumference of the healthy aorta; but when compared with its own ascending portion, it was one inch and three and a half lines less than it should have been to have carried out the same proportion. The ascending aorta was increased one inch in circumference beyond its natural dimensions, and when compared with the constricted portion just mentioned was very nearly one inch and eight and a half lines more than it should have been. Here, then, existed dilatation of all the coats of the ascending aorta, and at the junction of the arch and the descending portion, a constriction, accompanied by destruction of the internal and middle coats of the aorta, giving rise to a condition which, had the patient lived longer, must necessarily have terminated in false or sacculated aneurism.—*N. J. Med. and Surg. Reporter.*

